# NAD(P)H Quinone Oxidoreductase Protects TAp63γ from Proteasomal Degradation and Regulates TAp63γ-Dependent Growth Arrest

**DOI:** 10.1371/journal.pone.0011401

**Published:** 2010-06-30

**Authors:** Oshrat Hershkovitz Rokah, Ofer Shpilberg, Galit Granot

**Affiliations:** 1 Felsenstein Medical Research Center, Beilinson Hospital, Sackler School of Medicine, Tel Aviv University, Petah-Tikva, Israel; 2 Institute of Hematology, Beilinson Hospital, Rabin Medical Center, Sackler School of Medicine, Tel Aviv University, Petah-Tikva, Israel; Texas A&M University, United States of America

## Abstract

**Background:**

p63 is a member of the p53 transcription factor family. p63 is expressed from two promoters resulting in proteins with opposite functions: the transcriptionally active TAp63 and the dominant-negative ΔNp63. Similar to p53, the TAp63 isoforms induce cell cycle arrest and apoptosis. The ΔNp63 isoforms are dominant-negative variants opposing the activities of p53, TAp63 and TAp73. To avoid unnecessary cell death accompanied by proper response to stress, the expression of the p53 family members must be tightly regulated. NAD(P)H quinone oxidoreductase (NQO1) has recently been shown to interact with and inhibit the degradation of p53. Due to the structural similarities between p53 and p63, we were interested in studying the ability of wild-type and polymorphic, inactive NQO1 to interact with and stabilize p63. We focused on TAp63γ, as it is the most potent transcription activator and it is expected to have a role in tumor suppression.

**Principal Findings:**

We show that TAp63γ can be degraded by the 20S proteasomes. Wild-type but not polymorphic, inactive NQO1 physically interacts with TAp63γ, stabilizes it and protects it from this degradation. NQO1-mediated TAp63γ stabilization was especially prominent under stress. Accordingly, we found that downregulation of NQO1 inhibits TAp63γ-dependant p21 upregulation and TAp63γ-induced growth arrest stimulated by doxorubicin.

**Conclusions/Significance:**

Our report is the first to identify this new mechanism demonstrating a physical and functional relationship between NQO1 and the most potent p63 isoform, TAp63γ. These findings appoint a direct role for NQO1 in the regulation of TAp63γ expression, especially following stress and may therefore have clinical implications for tumor development and therapy.

## Introduction

p63, a p53 family member, is expressed from two different promoters generating two classes of proteins: TAp63, containing the N-terminal transactivation (TA) domain, and ΔNp63, lacking this domain. In addition, alternative splicing generates three different C-termini: α, β and γ. Given that the transactivation activity resides in the protein's N-terminus, TAp63 isoforms function as transcription factors inducing cell cycle arrest and apoptosis. TAp63γ is the most potent transcription activator [1], [2]. This isoform mimics p53 in culture and is capable of rescuing the growth inhibitory function of p53, in p53-deficient cells [3], [4]. These observations suggest that TAp63γ has tumor-suppressive properties analogous to p53. Supporting this notion is the fact that p63 maps to a chromosome region often deleted in cancers [5]. Likewise, loss of TAp63 expression has been detected in several cancers and has been associated with increased metastatic potential [6]–[8]. In opposition to the TA isoforms, ΔNp63 protects from apoptosis by directly competing for TAp63 target promoters [1], [9]. Over-expression of ΔNp63 isoforms observed in epithelial cancers suggests that p63 can also act as an oncogene [10]–[12]. However, the predominant physiological role of p63 is in epithelial development, as demonstrated by lack of epidermis and other epithelia in p63-deficient mice [13].

To avoid unwanted cell death accompanied by proper response to stress when needed, p53 family members have to be kept in check. In unstressed cells, p53 activity is restrained via the RING-type ubiquitin ligases Mdm2, Pirh2 and COP1. When cells encounter genotoxic stress, p53 protein levels rapidly increase. This correlates with a decrease in Mdm2 catalyzed poly-ubiquitylation and an increase in a variety of other post-translational modifications [14]. In contrast to the well-studied p53 protein, little is known about the molecular mechanisms regulating p63. Some evidence indicate that following genotoxic stress p63 is phosphorylated by kinases such as p38 MAP kinase resulting in stabilization of the TAp63 isoforms and detachment of ΔNp63α from p53-dependent promoters followed by its accelerated degradation [15]. Recent evidences have indicated a physical and functional relationship between p63 and Itch/AIP4, a HECT ubiquitin ligase. The data clearly indicate that all p63 isoforms are targeted by Itch for degradation [16]. In addition to the regulation of p63's degradation, crosstalk between p63 and proteins such as ASPP1/2 [17], PML [18], Sp1/3 [19] and p300 [20] has been shown to lead to increased transcriptional activity and stability of TAp63.

NAD(P)H quinone oxidoreductase (NQO1) is a cytosolic enzyme that catalyzes two-electron reduction of quinones, with NADH/NADPH as electron donors. NQO1 expression is induced in response to a variety of signals including oxidants and ionizing radiation. A C609T substitution, encoding for a Pro187Ser amino acid change is the major NQO1 polymorphism described. This polymorphic variant possesses less than 4% of the wild-type enzymatic activity and is associated with increased risk of developing different types of tumors [21]–[23]. Recently, ornithine decarboxylase, p33^ING1b^, p53 and p73 were found to be degraded by 20S proteasomes and NQO1 was shown to inhibit this degradation [24]–[29]. The finding that NQO1 supports the accumulation of p53 attributes to NQO1 a role as a tumor suppressor.

Due to the structural similarities between p53 and p63, it seemed plausible that NQO1 also regulates p63 expression. Our study tests this idea. We have chosen to focus on TAp63γ, as it is the most potent transcription activator and it is expected to have a role in tumor suppression. Our data demonstrate that wild-type but not C609T NQO1 binds to, stabilizes and inhibits 20S proteasomal degradation of TAp63γ. We further show that downregulation of NQO1 inhibits TAp63γ-dependant p21 upregulation and TAp63γ-induced growth arrest stimulated by doxorubicin. These findings provide insight into the contribution of NQO1 to p63 stability.

## Materials and Methods

### Cell lines

HCT116 (kindly provided by Prof. Yossi Shaul, Weizmann Institute of Science, Israel), HCT116^−/−^ (kindly provided by Prof. Moshe Oren, Weizmann Institute of Science, Israel), HEK293 cells (kindly provided by Prof. Yehiel Zick, Weizmann Institute of Science, Israel) were maintained in DMEM supplemented with 10% serum, 2mM glutamine, penicillin/streptomycin and cultured at 37°C in a humidified incubator with 5% CO_2_.

### Compounds

Doxorubicin (DOX) (Sigma) was dissolved in H_2_O; 1µM was added to the cells, unless stated otherwise. The proteasome inhibitor, MG132 (Sigma), was dissolved in DMSO. Cyclohexamide (Sigma), 10µg/ml was added to the cells.

### Plasmids and cloning

Wild-type NQO1 (GenBank accession no. J03934) was cloned into an HA-pCDNA3 expression vector. C609T NQO1 was generated using the QuikChange site-directed mutagenesis kit according to the manufacturer's instructions (Stratagene) with appropriate primers:

Sense: 5′-GTGGCTTCCAAGTCTTAGAATCTCAACTGACATATAGC-3′.

Anti-sense: 5′-GCTATATGTCAGTTGAGATTCTAAGACTTGGAAGCCAC-3′.

Flag-tagged NQO1 expression vector was kindly provided by Prof. Yosef Shaul, Weizmann Institute of Science, Israel. TAp63γ expression vector was kindly provided by Prof. Kurt Engeland, University of Leipzig, Germany.

### siRNA transfections

siRNA oligonucleotides targeting NQO1 or scrambled oligonucleotides (Ambion) were transfected using siPORT NeoFX (Ambion) following manufacturer's guidelines. After 24h, cells were transfected with TAp63γ as described below, and incubated for an additional 24h before being harvested.

### Transfections

Transfections of TAp63γ and NQO1 expression plasmids were performed using jetPEI transfection reagent (Polyplus-transfection), following manufacturer's guidelines. Briefly: Cells were seeded in 6-well plates 24h before transfection. JetPEI was mixed with each plasmid and complex formation was allowed to take place for 20 min at room temperature before being added to the wells. Cells were harvested 48h later, as described below. Stable transfection of HA-NQO1 in 293 cells was performed in the same manner followed by neomycin-selection. Neomycin-resistant colonies expressing HA-NQO1 were identified by immunoblot analysis with anti-HA antibody.

### Western blot

Cells were harvested using lysis buffer (50mM Tris-HCl pH 7.4, 150mM NaCl, 1mM EDTA, 1% TRITON X-100 and protease inhibitor cocktail). Equal amounts of protein were separated by 10% SDS-PAGE and blotted onto PVDF membranes. Membranes were blocked over night at 4°C and probed with the appropriate primary for 1h at room temperature and then with the appropriate fluorescently-labeled secondary antibody (Li-Cor Biosciences). Membranes were scanned using ODYSSEY Infrared Imaging System (Li-Cor Biosciences). Primary antibodies used: NQO1, p63 (4A4), GAPDH (Santa Cruz Biotechnology); HA (Covance); FLAG (Sigma).

For stabilization experiments, cells were transfected with TAp63γ and NQO1 expression plasmids as described above. Twenty-four hours after the transfections, the cells were treated with 10µg/ml cyclohexamide for 4h. Cells were then collected and analyzed by Western blot analysis as described.

### Real-time PCR

RNA was isolated using RNAqueous-4PCR kit (Ambion) and reverse transcribed using the high capacity cDNA RT kit (Ambion). Real-time PCR was then performed using the SDS 7000 machine (Applied Biosystems) in a 20µl reaction containing 40ng RNA, 10µl TaqMan master mix (Ambion), 1µl of target gene or 18S rRNA control primers and a FAM dye-labeled TaqMan probe (Ambion). Amplification conditions were: 50°C for 2 min, followed by 95°C for 10 min, then 40 cycles of 95°C for 15 sec and 60°C for 1 min. The ΔΔC_t_ method was used to calculate relative expression levels.

### Reverse transcription (RT)-PCR

RNA was extracted using Nucleospin RNA II kit (Macherey-Nagel) and reverse transcribed with RevertAid M-MuLV (Fermentas). PCR reactions were performed using TAp63 primers and gapdh control primers. TAp63 PCR products were separated on agarose gels and their intensity was calculated relative to the gapdh PCR products, using ImageJ. Primers used: TAp63 forward 5′-TCGTAGAAACCCCAGCTCAT-3′; reverse 5′-TTGTTTGTCGCACCATCTTC-3′. gapdh forward 5′-ACCACAGTCCATGCCATCAC-3′; reverse: 5′-CCACCACCCTGTTGCTGTA-3′.

### Cell cycle analysis

Cells were transfected with either siNQO1 oligonucleotides or scrambled oligonucleotides. Twenty-four hours following this transfection, the cells were transfected with a TAp63γ expression plasmid (as described above). Cells were treated with 0.05µM DOX 24h after the transfections. Following an additional 24h, the cells were collected and fixed in 70% ethanol. Nuclei of fixed cells were prepared for analysis using a detergent-trypsin method followed by staining with propidium-iodide [30]. DNA content was analyzed by FACSCALIBUR (Becton Dickinson), using ModFitLT cell cycle analysis software (Verity Software House Inc.).

### Co–immunoprecipitation

Immunoprecipitation (IP) was carried out using the ExactaCruz product (Santa Cruz Biotechnology) following manufacturer's guidelines. Briefly, Cells were transfected with TAp63γ and NQO1 expression plasmids, as described above. Following 48h, cells were lysed with 500µl lysis buffer (50mM Tris-HCl pH 7.4, 150mM NaCl, 1mM EDTA, 1% TRITON X-100 and protease inhibitor cocktail) and immunoprecipitated with Exactacruz IP-matrix that was previously conjugated to anti–HA or anti-p63 antibodies. Immunoprecipitated samples were Western blot analyzed using appropriate primary antibodies and the secondary antibodies supplied by the ExactaCruz product.

### In-vitro translation

TAp63γ and NQO1 expression plasmids were transcribed and translated using the TnT®-T7 Coupled Reticulocyte Lysate System (Promega) and the Transcend® Non-Radioactive Translation Detection Systems (Promega) for incorporating biotinylated lysine residues into proteins during translation, as described by the supplier. One µl Transcend® tRNA and 1µg of plasmid DNA were routinely used in a 50µl assay. Reactions were incubated at 30°C for 90 min.

### In-vitro protein degradation assays

Degradation of in-vitro translated, biotin-labeled p63 with 1µg of purified 20S proteasome (ABR-Affinity BioReagents) was carried out in degradation buffer (100mM Tris-HCL (pH 7.5), 150mM NaCl, 5mM MgCl, 2mM DTT), with or without in-vitro translated, biotin-labeled NQO1 and 1mM NADH, at 37°C for 4h. Samples were then electrophoresed on SDS-PAGE and transferred to PVDF membranes. Membranes were incubated with fluorescently-labeled streptavidin for 30 min at room temperature and scanned using ODYSSEY Infrared Imaging System (Li-Cor Biosciences).

### Statistics

Experiments were done in triplicates. Student's *t*-test was used where indicated. p<0.05 was considered significant.

## Results

### Wild-type but not C609T NQO1 stabilizes TAp63γ expression

**Figure 1:**
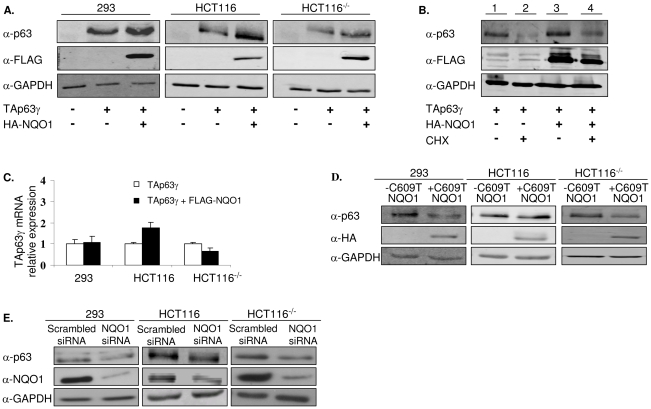
TAp63γ protein is stabilized by NQO1. (A) 293, HCT116 and HCT116^−/−^ cells expressing endogenous NQO1 or over-expressing wild-type FLAG-NQO1 were transfected with a plasmid expressing TAp63γ. Forty-eight hours post-transfection cell lysates were prepared and resolved by SDS-PAGE. TAp63γ, NQO1 and GAPDH levels were detected by Western blot analysis using anti-p63 (4A4), anti-FLAG and anti-GAPDH (loading control) antibodies, respectively. (B) HCT116 cells expressing endogenous NQO1 or over-expressing wild-type FLAG-NQO1 were transfected with a plasmid expressing TAp63γ. Twenty-four hours post transfection, 10µg/ml cyclohexamide (CHX) was added for 4 hours. Cell lysates were prepared and resolved by SDS-PAGE. TAp63γ, NQO1 and GAPDH levels were detected by Western blot analysis using anti-p63 (4A4), anti-FLAG and anti-GAPDH (loading control) antibodies, respectively. (C) RNA was prepared from these same cells, reverse transcribed and RT-PCR was performed using primers specific for TAp63 and for gapdh. Data is represented as relative levels of TAp63γ normalized to gapdh. (D) 293, HCT116 and HCT116^−/−^ cells were co-transfected with plasmids expressing TAp63γ and HA-C609T NQO1. Forty-eight hours post-transfection cell lysates were prepared and resolved by SDS-PAGE. TAp63γ, NQO1 and GAPDH levels were detected by Western blot analysis using anti-p63 (4A4), anti-HA and anti-GAPDH (loading control) antibodies, respectively. (E) 293, HCT116 and HCT116^−/−^ cells were transfected with scrambled oligonucleotides or siNQO1 oligonucleotides. Twenty-four hours post-transfection cell were transfected with a plasmid expressing TAp63γ. Twenty-four hours post-transfection cell lysates were prepared and resolved by SDS-PAGE. TAp63γ, NQO1 and GAPDH levels were detected by Western blot analysis using anti-p63 (4A4), anti-NQO1 and anti-GAPDH (loading control) antibodies, respectively.

The observation that NQO1 stabilizes p53 raised the question whether NQO1 also participates in dictating p63 expression level. To clarify this issue, we cotransfected FLAG-tagged NQO1 and TAp63γ expression plasmids into HCT116 and 293 cells. Both these cell lines do not express detectable levels of endogenous TAp63γ (Fig. 1A). As predicted, both HCT116 and 293 cells over-expressing NQO1 showed an increase in TAp63γ protein level (Fig. 1A). In order to test whether TAp63γ stabilization by NQO1 is dependent on p53, these transfections were also conducted in p53 null, HCT116^−/−^ cells. These cells do not express detectable levels of endogenous TAp63γ either (Fig. 1A). Once again, NQO1 over-expression resulted in elevated TAp63γ protein levels, suggesting that this effect is independent of the cell's p53 status (Fig. 1A). In order to confirm that NQO1 indeed prolongs TAp63γ stability, the degradation rate of TAp63γ in HCT116 cells treated with cyclohexamide was compared to that of TAp63γ in the presence of NQO1 in HCT116 cells treated with cyclohexamide. TAp63γ levels decreased significantly following cyclohexamide treatment and by 4 hours were nearly down to 20% compared with non-treated cells (Fig. 1B compare lanes 1, 2). In contrast, the level of TAp63γ in the presence of NQO1 was only moderately decreased, to 60%, following cyclohexamide treatment compared with non-treated cells (Fig. 1B compare lanes 3, 4). Similar results were documented in HCT116^−/−^ cells, once again implying that this effect is independent of the cell's p53 status (data not shown). No upregulation was observed in TAp63γ mRNA level under these conditions in 293 and HCT116^−/−^ cells. A slight upregulation in TAp63γ mRNA expression was detected in HCT116 cells (Fig. 1C). To determine whether the enzymatic activity of NQO1 is required for p63 stability, we transfected an HA-tagged C609T NQO1 expression plasmid into all 3 cell lines. Unlike wild-type NQO1, C609T NQO1 did not stabilize cotransfected TAp63γ (Fig. 1D). Of note, the expression of transfected C609T NQO1 in these cells was lower than that of transfected wild-type NQO1 (Fig. 1A and 1D, middle panels). Continuous attempts to increase C609T NQO1 expression level, in transient and even in stably transfected lines, failed. This is possibly due to the fact that the mutant protein is considered to be unstable [31]. In a paper published by Traver et al [32], the specific activity of C609T NQO1 purified protein from E. coli cells was 2% of the specific activity of the wild-type recombinant protein probably due to diminished ability to bind FAD. The authors state however that according to their data an additional possible reason for the lack of enzymatic activity could be to be due to very poor expression of the C609T NQO1 protein. These data therefore indicate that TAp63γ is stabilized by NQO1 and that NQO1 enzymatic activity may be required for this.

### Effect of NQO1 silencing on TAp63γ stability

Dicumarol is a common NQO1 inhibitor. However, dicumarol was shown to be nonspecific, inhibiting several quinone reductases and having many ancillary effects [33], [34]. Consequently, we decided to inhibit NQO1 by using NQO1 specific siRNA molecules. HCT116 and 293 cells were transfected with siNQO1 oligonucleotides or with scrambled oligonucleotides and with a TAp63γ expression plasmid. Expression of NQO1 mRNA and protein, in the presence of siNQO1, was reduced by an average of 99% and >60% as detected by real-time PCR and Western blot, respectively (data not shown and Fig. 1E). In both cell lines, reduction in NQO1 caused a decrease in TAp63γ expression (Fig. 1E). A similar outcome of NQO1 silencing on p63 expression level was observed in HCT116^−/−^ cells, suggesting once again, that this effect is independent of the cell's p53 status (Fig. 1E). These data indicate that decreased NQO1 expression leads to downregulation of TAp63γ.

### TAp63γ stabilization by NQO1 is enhanced under stress

**Figure 2:**
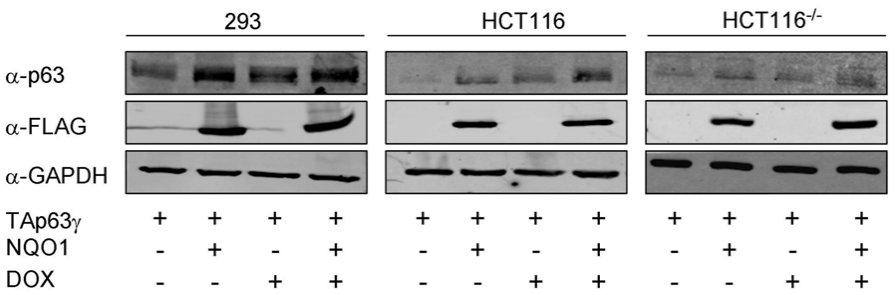
TAp63γ expression is stabilized by NQO1 in response to genotoxic stress. 293, HCT116 and HCT116^−/−^ cells expressing endogenous NQO1 or over-expressing wild-type FLAG-NQO1 were transfected with a plasmid expressing TAp63γ. Twenty-four hours post-transfection cells were treated with 1µM DOX for 24h. At this point, cell lysates were prepared and resolved by SDS-PAGE. TAp63γ, NQO1 and GAPDH levels were detected by Western blot analysis using anti-p63 (4A4), anti-FLAG and anti-GAPDH (loading control) antibodies, respectively.

Under normal growth conditions, TAp63γ protein level is elevated in cells over-expressing NQO1 (Fig. 1A). p63 expression is known to be induced following stress [35]. At this point, we were interested in determining the effect of NQO1 on TAp63γ following stress. To this end, cells co-transfected with TAp63γ and NQO1 expression plasmids were treated with DOX. We show that the stabilizing effect of NQO1 on TAp63γ was more prominent after induction of stress by DOX exposure (Fig. 2). In contrast, C609T NQO1 did not lead to elevated expression of TAp63γ following DOX treatment (data not shown). These findings suggest that NQO1 stabilizes TAp63γ to a higher extent in stressed cells as compared to unstressed cells and that NQO1 enzymatic activity may be required for this.

### Wild-type but not C609T NQO1 physically interacts with TAp63γ

**Figure 3:**
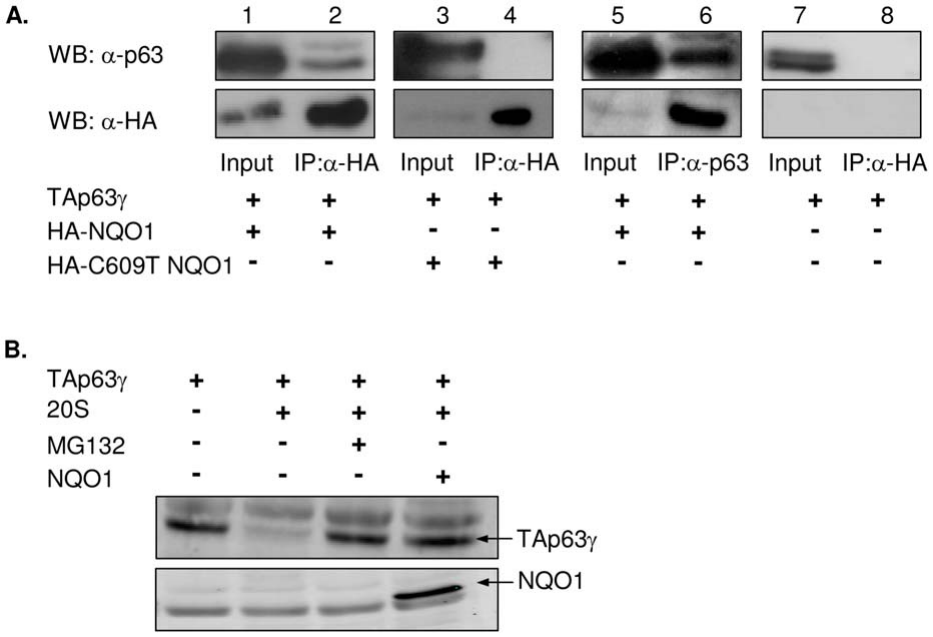
NQO1 physically associates with TAp63γ and protects it from 20S proteasomal degradation. (A) 293 cells stably expressing HA-NQO1 or HA-C609T NQO1 were transfected with a plasmid expressing TAp63γ. Cellular extracts were prepared and subject to immunoprecipitated (IP) with anti-HA or anti-p63 antibodies. Immunoprecipitated proteins and 5% of input material were detected by Western blot using anti-p63 and anti-HA antibodies. (B) Degradation of in-vitro translated, biotin-labeled TAp63γ with 20S proteasome was carried out in the presence or absence of in-vitro translated, biotin-labeled NQO1 and 1mM NADH at 37°C for 4h. Biotin-labeled TAp63γ and NQO1 were detected with fluorescently-labeled streptavidin.

To detect a potential physical association between TAp63γ and NQO1, 293 cells stably expressing HA-NQO1 were transfected with a TAp63γ expression plasmid. Fig. 3A demonstrates that immunoprecipitation of HA-NQO1 pulled down TAp63γ (lane 1–2). In agreement, immunoprecipitation of TAp63γ pulled down HA-NQO1 (lane 5–6). Conversely, immunoprecipitation of C609T NQO1 did not pull down this p63 isoform under the same conditions (Fig. 3A, lanes 3–4). These data confirm that TAp63γ and NQO1 do interact physically and that this interaction may be dependent on the catalytic activity of NQO1.

### NQO1 protects TAp63γ from 20S proteasomal degradation

To study whether NQO1 protects TAp63γ from degradation, an in-vitro degradation assay was performed. In this assay, in-vitro translated TAp63γ was used as a substrate for degradation by 20S proteasomes in the absence or presence of in-vitro translated NQO1. As has previously been shown for p53 and p73 [26]–[29], TAp63γ was degraded by 20S proteasomes. This degradation was inhibited in the presence of excess NQO1 and its cofactor, NADH, suggesting that NQO1 directly protects TAp63γ from degradation by 20S proteasomes (Fig. 3B). Unlike wild-type NQO1, C609T NQO1 did not inhibit this degradation (data not shown). It should be noted that the expression level of C609T NQO1 in the presence of 20S proteasomes was very low compared to wild-type NQO1. It is impossible to establish, at this point, whether the absence of inhibition of TAp63γ degradation by C609T NQO1 was due to the incompetence of C609T NQO1 or was it merely a result of its low expression level.

### Downregulation of NQO1 inhibits TAp63γ-mediated growth arrest

**Figure 4:**
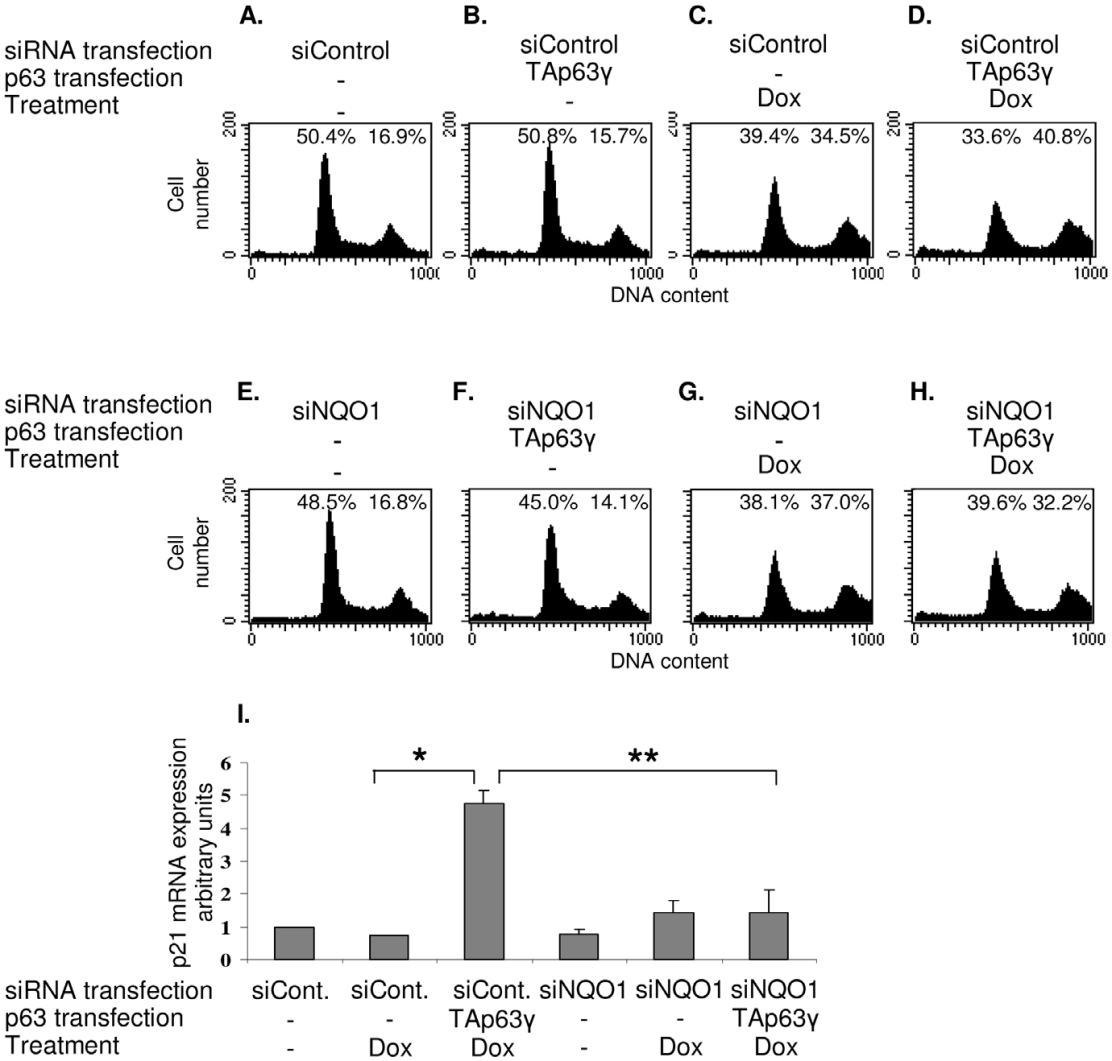
Downregulation of NQO1 inhibits TAp63γ-dependent growth arrest in DOX treated HCT116^−/−^ cells. HCT116^−/−^ cells were transfected with scrambled oligonucleotides (siControl) (A–D) or siNQO1 oligonucleotides (E–H) and transfected (B, D, F, H) or not (A, C, E, G) with a plasmid expressing TAp63γ. Twenty-four hours post-transfection the cells were treated or not with 0.05µM DOX. 24h after this treatment, DNA content was analyzed by propidium-iodide staining. The percentage of cells in G1 and G2 is presented. (I) RNA was prepared from these same cells, reverse transcribed and real time PCR was performed using primers specific for p21 and 18S rRNA. Data is represented as relative levels of p21 normalized to 18S rRNA. *p<0.05, **p<0.01.

We next determined whether NQO1-dependant TAp63γ accumulation could initiate growth arrest or apoptosis. In order to neutralize the effect of p53, p53 null HCT116^−/−^ cells were used. Since HCT116 cells tend to undergo growth arrest rather than apoptosis we tested the effect of NQO1 on TAp63γ-mediated growth arrest. Exposure of HCT116^−/−^ cells to DOX resulted in G2 arrest (Fig. 4C). The presence of TAp63γ lead to a slight increase in G2 arrested cells (Fig. 4D). A 5-fold increase in p21 mRNA expression was also detected in these TAp63γ over-expressing cells exposed to DOX in comparison to cell exposed to DOX but not expressing TAp63γ (Fig. 4I). NQO1 inhibition in DOX treated, TAp63γ transfected cells, resulted in a reduction of G2 arrested cells to the level observed following DOX in cells not expressing TAp63γ (Fig. 3H). Accordingly, TAp63γ and DOX-mediated increase in p21 level was almost completely reversed by siNQO1 (Fig. 3I). Expression of TAp63γ, without an additional stress signal, did not lead to cell cycle arrest in our setting. These results are consistent with our NQO1 overexpression and silencing data and suggest that NQO1 is an important regulator of TAp63γ and consequently of p21. Our data indicate that downregulation of NQO1 inhibits TAp63γ-mediated p21 upregulation and TAp63γ-induced G2 arrest in DOX treated HCT116^−/−^ cells.

## Discussion

Similar to p53, TAp63γ protein level increases upon treatment with DNA damaging agents resulting in transactivation of p53-responsive genes assigning p63 as an important component of the cell's apoptotic machinery [35]–[37]. This p63 upregulation is not the result of transcriptional activation, but is most likely due to an increase at the protein level [36], [37]. To date, little is known of the molecular mechanisms regulating p63 protein level. We provide in-vitro evidence for a physical and functional relationship between TAp63γ and NQO1 supporting a new pathway regulating p63 stability.

In different types of unstressed cells, wild-type NQO1 bound to and stabilized TAp63γ. In contrast, we show that C609T NQO1 is unable to associate with TAp63γ or to affect its protein level. This observation emphasizes the requirement of proper NQO1 enzymatic activity for p63 stabilization. It remains to be determined whether intact enzymatic activity of NQO1 and its binding to TAp63γ is sufficient for p63 stabilization. NQO1 did not alter the expression of GAPDH or retinoblastoma (data not shown for retinoblastoma) implying that this protein degradation regulatory pathway is specific to certain proteins.

p63 has been shown to undergo ITCH mediated 26S proteasome degradation [16]. Our results show that, in addition, TAp63γ is subjected to 20S proteasomal degradation and that NQO1 protects TAp63γ from such degradation. This finding implies that p63 stabilization may involve several parallel mechanisms.

Many evidence support the notion that p63 has tumor suppressor functions: 1) p63 specific siRNA enhances the transformation potential of p53^−/−^ MEFs [38]; 2) p63 can mediate chemo-sensitivity independent of p53 status by induction of apoptosis [17], [39]; 3) The combined absence of p63 and p73 severely impairs the induction of p53-dependent apoptosis in response to DNA damage [40]. These observations suggest that p63 may complement, and in some circumstances substitute for p53. For these reasons complex molecular mechanisms must regulate the expression of the p63 isoforms. Phosphorylation has been reported to be associated with rapid accumulation of TAp63γ upon genotoxic treatment whereas ΔNp63α is degraded under these same conditions [15]. Coherently, in our settings, NQO1 over-expression accompanied by stress (DOX) resulted in the accumulation of TAp63γ and not in the accumulation of the antagonizing ΔN isoforms or the weak TAp63γ (data not shown). Stabilization of TAp63γ by NQO1 is of biological relevance since the ability of TAp63γ to upregulate p21 and to induce G2 arrest in stressed HCT116^−/−^ cells is almost abolished in the presence of siNQO1.

Our results have biological implications concerning the understanding of tumor development. Cells carrying polymorphic inactive NQO1 that are exposed to carcinogens that are substrate for detoxification by NQO1 will accumulate more damage than cells carrying wild-type NQO1. Our results show that polymorphic NQO1 does not stabilize TAp63γ and low wild-type NQO1 levels (as detected in cells harboring one allele of polymorphic NQO1) decrease TAp63γ-induced growth arrest. These cells, already harboring damaged DNA, would therefore also exhibit imperfect apoptotic or growth arrest responses, providing them with a growth advantage that could potentially lead to tumor development. Indeed, p63 is not frequently mutated in cancer but rather alterations of p63 expression have been widely reported [10]–[12], [41]. Of note, C609T NQO1 is known to enhance susceptibility to bladder and breast cancers [21]–[23]. In these same tumor types, TAp63 expression is often misregulated [6]–[8]. The altered TAp63 expression levels detected in these tumors may be explained by the incompetence of C609T NQO1 to properly stabilize p63. In one study, Fagerholm et al demonstrated that the chemotherapeutic response of C609T homozygous breast carcinoma cells was impaired in p53-aberrant tumors [21]. This phenomenon could be attributed to the inability of polymorphic NQO1 to stabilize TAp63, whose expression was indeed shown to be significantly reduced in breast cancers. TAp63 reduction could result in the poor chemotherapeutic response characteristic of these tumor cells.

Our report is the first to identify a new mechanism appointing a direct role for NQO1 in the regulation of p63 expression, especially following stress. We propose that NQO1 associates with and stabilizes TAp63γ. The increase in TAp63γ results in enhanced transcription of p21 consequently leading to growth arrest. Characterization of the interactions between NQO1 and the other p63 family members would be an interesting future study. Such studies will probably elucidate that NQO1 not only regulates the expression of the potent TAp63γ but more realistically it regulates the balance of p63 isoforms under normal growth conditions and following stress. It will be interesting to explore how this level of regulation collaborates with others as more regulators of p63 are found. Clearly, comprehension of such regulations of the p63 isoforms will lead to a greater understanding of the role of p63 in tumor suppression.
